# Prognostic potential of nutritional risk screening and assessment tools in predicting survival of patients with pancreatic neoplasms: a systematic review

**DOI:** 10.1186/s12937-024-00920-w

**Published:** 2024-02-03

**Authors:** Mengxia Yu, Xiaoxuan Li, Mingxia Chen, Linglong Liu, Tianying Yao, Jiarong Li, Wang Su

**Affiliations:** School of Nursing, Nanjing Medical University, 818 Tianyuan East Road, Jiangning District, Nanjing, 211166 Jiangsu Province China

**Keywords:** Pancreatic neoplasms, Nutritional screening, Nutritional assessment, Overall survival, Prognosis

## Abstract

**Backgrounds & Aims:**

The nutritional evaluation of pancreatic cancer (PC) patients lacks a gold standard or scientific consensus, we aimed to summarize and systematically evaluate the prognostic value of nutritional screening and assessment tools used for PC patients.

**Methods:**

Relevant studies were retrieved from major databases (PubMed, Embase, Web of Science, Cochrane Library) and searched from January 2010 to December 2023. We performed meta-analyses with STATA 14.0 when three or more studies used the same tool.

**Results:**

This analysis included 27 articles involving 6,060 PC patients. According to a meta-analysis of these studies, poor nutritional status evaluated using five nutritional screening tools Prognostic Nutritional Index (PNI), Geriatric Nutritional Risk Index (GNRI), Controlling Nutritional Status Score (CONUT), Nutrition Risk Screening (NRS2002) and Glasgow Prognostic Score (GPS) was associated with all-cause mortality in PC patients. But Modified Glasgow Prognostic Score (mGPS) did not. Of all tools analyzed, CONUT had the maximum HR for mortality (HR = 1.978, 95%CI 1.345–2.907, *P* = 0.001).

**Conclusion:**

All-cause mortality in PC patients was predicted by poor nutritional status. CONUT may be the best nutritional assessment tool for PC patients. The clinical application value of Short Form Mini Nutritional Assessment (MNA-SF), Generated Subjective Global Assessment (SGA) and Patient-generated Subjective Global Assessment (PG-SGA) in PC patients need to be confirmed. In order to improve patients’ nutritional status and promote their recovery, nutritional screening tools can be used.

**Registration:**

This systematic review was registered at the International Prospective Register of Systematic Reviews (PROSPERO) (number CRD42022376715).

## Introduction

Pancreatic cancer (PC), a group of malignant tumors mainly originating from pancreatic duct- epithelium and acinus cells, is one of the most common malignant tumors in the digestive system [1]. The global prevalence of pancreatic cancer is about 2.6% [2]. Over the past 25 years, the global burden of pancreatic cancer has doubled and now ranks in the top 10 of all cancers in more than 130 countries [3]. With the progression of pancreatic cancer, malnutrition has become the most common and difficult problem for pancreatic cancer patients [4]. On the one hand, pancreatic cancer patients lead to tumor cachexia due to abnormal pancreatic secretion function and increased tumor metabolism. On the other hand, the anatomical changes caused by digestive tract reconstruction after tumor resection often lead to patients’ decreased appetite and difficulty in eating, and thus malnutrition [5]. Yu K [6]investigated 687 tumor patients, and the highest nutritional risk was pancreatic cancer (81.8%). Nutritional status affects the incidence of postoperative complications, length of hospital stay, and long-term prognosis of cancer patients [7–10]. Malnutrition has been proven to be an independent risk factor for prognosis in patients with pancreatic cancer [11]. Therefore, nutritional risk screening and nutritional support should be conducted before pancreatic cancer resection, and early identification and intervention of malnourished patients can indeed reduce postoperative complications, thus shortening hospital stay and reducing hospital costs [11].

Despite the nutritional abnormalities, most patients did not receive nutritional advice before undergoing chemotherapy despite their nutritional deficiencies [12, 13]. It is of significant importance for patients to obtain nutritional advice from all members of their medical team, in case a dietitian is unavailable. European Society for Clinical Nutrition and Metabolism recommends that cancer patients should undergo long-term repeated nutritional screening to identify patients at risk of malnutrition [14]. Therefore, the application of nutritional screening and assessment tools to assess preoperative nutritional status of pancreatic cancer patients, early detection of malnutrition risk and appropriate interventions can improve clinical outcomes [15].

Research and clinical experience continue to provide us with new tools for nutritional screening and assessment, providing us with more options for evaluating the prognosis of pancreatic cancer [16–18]. The prevalence of malnutrition in PC differed greatly from previous studies. As a result, many nutritional assessment or screening tools are employed [19]. The prevalence of malnutrition in PC patients differed from 9.1% (by CONUT) to 39.7% (by PNI) in the same cohort [20]. . In PC patients, this makes tracking prevalence and comparing the effects of different nutrition management interventions challenging. Thus, this study aims to provide a reference for the selection and evaluation of nutritional evaluation tools based on the prognostic value.

## Method

### Search strategy and selection criteria

Studies were retrieved from major databases (PubMed, Embase, Web of Science, Cochrane Library) and searched from the earliest available date until October. This systematic review was registered at the International Prospective Register of Systematic Reviews (PROSPERO) (number CRD42022376715). Inclusion criteria were as follows: Inclusion criteria were as follows: (1) P (patients): The participants were patients with PC (≥ 18 years old); (2) I (intervention—exposure): patients with malnutrition risk as determined by ESPEN 2017 recommended tools; (3) C (control): patients with a normal nutritional status as determined by ESPEN 2017 recommended tools [14]; and (4) O (outcomes): studies that reported all-cause mortality. A cut-off value to divide patients into malnutrition and normal nutrition groups was identified for (2) and (3). The hazard ratios (HRs) and corresponding 95% confidence intervals (CIs) for (4) were either directly reported by the studies or could be calculated using the data provided. The exclusion criteria included: (1) Research proposals, guidelines, conference abstracts, reviews; (2) Studies not published in English or Chinese; (3) Full text not available. The following search terms were used: “Pancreatic Neoplasms“[MeSH Terms] AND (“prognos*“[Title/Abstract] OR “predict*“[Title/Abstract] OR “mortality“[Title/Abstract] OR “survival“[Title/Abstract]) AND (“malnutri*“[Title/Abstract] OR “nutri*“[Title/Abstract] OR “undernutri*“[Title/Abstract]). Search database from search date January 2010 to December 2023. Additionally, reference lists of the cited articles were manually searched to identify additional relevant articles.

### Screening of the articles

Using the database returned articles’ titles and abstracts, two investigators (Yu & Li) independently searched the database. To determine if studies met the inclusion criteria, the full text of the studies was read. The inclusion discrepancies between the other two investigators were confirmed by a third investigator (Liu).

### Quality assessment of the articles

Cohort studies were evaluated using the modified Newcastle-Ottawa Scale (NOS) [21], in which 1 to 3, 4 to 6, and 7 to 9 scores were considered low, medium, and high quality, respectively. Studies with a final score above 6 were considered high quality.

### Data extraction

Full texts of the screened articles were carefully reviewed and data were extracted: surname of the first author, publication year, study design (retrospective or prospective), country, disease stage, sample size, mean/median age or age range, categorical or continuous analysis of nutritional status score, most fully adjusted risk estimate, follow-up duration, determination method and cut-off value of nutrition evaluation tool, therapeutic method. Each study’s hazard ratio (HR) and 95% Confidence Interval (CI) were directly extracted from the multivariate analysis.

### Statistical analysis

The meta-analysis was conducted using STATA 14.0. We acquired hazard ratios with 95% confidence intervals for each study, and then plotted the pooled results. Statistical analyses were performed if three or more studies used the same nutrition screening or assessment tool. Heterogeneity was explored using *I*^*2*^ statistics and Cochran’s Q test. The determination of significant high heterogeneity in studies was based on the criteria of *I*^*2*^ ≥ 50% or *P*<0.10 of the Cochran Q test, after which a random-effects model was employed [22]. Conversely, studies exhibiting *I*^*2*^<50% and *P*>0.10 were analyzed using a fixed-effects model. Heterogeneity among studies was considered minor if *I*^*2*^<25%, moderate if *I*^*2*^ values ranged between 25% and 50%, and large if *I*^*2*^ values exceeded 50%. Statistical significance was established at a *P* value less than 0.05.

## Results

### Literature search

**Fig. 1:**
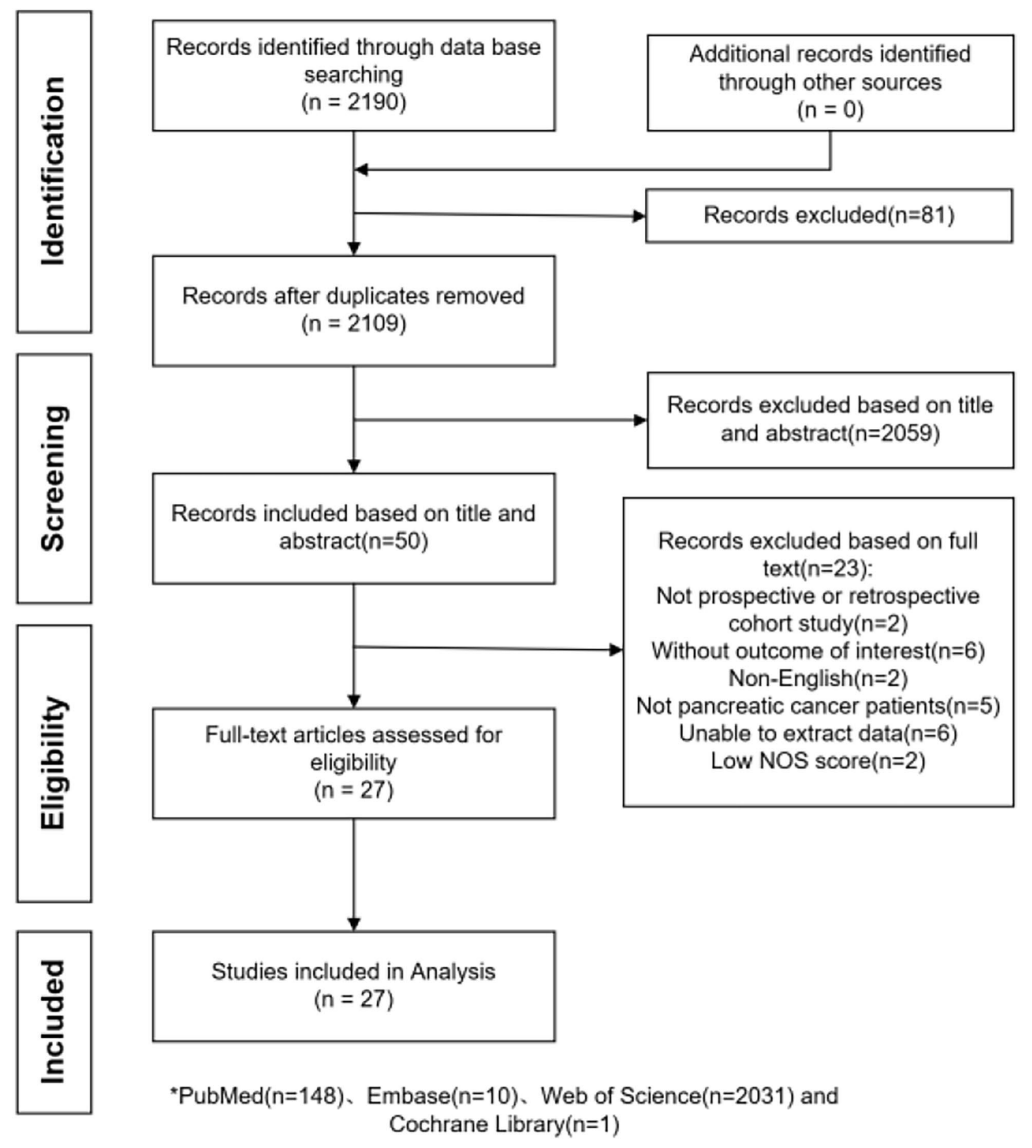
Study selection process

In total, 2189 references were identified, including 148 in PubMed, 10 in Embase, 2031 in Web of Science, and 1 in Cochrane Library. According to the manual analysis of these studies, 27 articles were included in the analysis. Meta-analysis was performed by 26 articles and qualitative analysis was performed by 2 articles. One article provided qualitative and quantitative analysis. Because multiple tools used in this article. The search and selection process is illustrated in Fig. 1.

### Study characteristics

The features of the 27 studies were outlined in Table 1. There were a total of 6,060 PC patients from either China, Japan, US, Korea, Norway, Italy, Turkey, Germany. A score of 6 to 9 was assigned to the study quality by NOS.

**Table 1:** Main characteristic of the included studies

Author/year	Region	Patients, n=	Age	Tool	Cut-off value of nutritional status	Undernutrition degree(%)	HR	95%CI	Follow-up (years)	Tumor stage	Therapeutic method	Study design	NOS
Abe 2021 [23]	Japan	159	——	GPS	0 vs. 1,2	22.0	0.613	1.146–2.208	5	I-II	Surgery	R	7
				PNI	≤ 40 vs. >40	19.5	1.330	0.701–2.524					
Asama 2018 [24]	Japan	72	63(42–85)	mGPS	0,1 vs. 2	12.5	26.160	5.220–131.100	2.4	III-IV	Chemotherapy	R	6
				PNI	<45.2 vs. ≥ 45.2	54.2	0.520	0.230–1.190					
				CONUT	<3 vs. ≥ 3	36.4	0.690	0.290–1.670					
Asaoka 2016 [25]	Japan	46	67	PNI	<47 vs. ≥ 47	45.7	1.216	0.420–3.750	3	I-II	Surgery	R	9
Geng 2015 [26]	China	321	60.0 ± 9.5	PNI	<47.3 vs. ≥ 47.3	46.4	0.627	0.453–0.868	2.6	III-IV	Chemotherapy	R	8
Itoh 2021 [19]	Japan	589	71(63–77)	CONUT	<3 vs. ≥ 3	30.39	1.236	0.925–1.653	5	I-III	Surgery	R	8
				PNI	<46 vs. ≥ 46	——	1.432	1.069–1.918					
				GPS	0,1 vs. 2	5.8	0.760	0.556–1.040					
				mGPS	0,1 vs. 2	9	1.157	0.738–1.813					
				GNRI	<103.77 vs. ≥ 103.77	——	0.916	0.683–1.229					
Kurahara 2015 [27]	Japan	96	——	GPS	0,1vs 2	——	4.673	1.802–12.110	7.7	IV	Chemoradiotherapy	R	8
				PNI	<45 vs. ≥ 45	——	1.256	0.729–2.169					
Lee 2017 [28]	US	499	62.1 ± 10.2	PNI	<49.5 vs. ≥ 49.5	41.7	1.562	1.240–1.967	9	I-IV	Surgery / Chemotherapy / Radiotherapy / No treatment	R	8
		121	62.9 ± 9.5	PNI	<49.5 vs. ≥ 49.5	——	1.645	1.020–2.654	9	I-II			
		260	62.7 ± 10.2	PNI	<49.5 vs. ≥ 49.5	——	1.573	1.189–2.081	2.6	IV			
Onoe 2021 [29]	Japan	187	68(36–85)	PNI	<36 vs. ≥ 36	33.2	1.600	1.110 − 2.300	5	I-IV	Surgery / Palliative surgery	R	8
Wang 2012 [30]	China	91	——	mGPS	0 vs. 1 vs. 2	——	1.264	0.680–2.349	4.8	I-IV	Surgery / Chemotherapy	R	7
Abe 2018 [31]	Japan	329	67(61–74)	PNI	<45 vs. ≥ 45	62.6	0.437	0.305–0.631	——	I-III	Surgery	R	8
				GPS	0 vs. 1,2	25.5	0.615	0.366–1.008					
				mGPS	0 vs. 1,2	14.3	1.258	0.726–2.220					
		95	65(58–69)	GPS	0 vs. 1,2	49.5	1.279	0.710–2.342	——	IV	Palliative surgery	R	8
				PNI	<45 vs. ≥ 45	34.7	0.719	0.388–1.349					
Park 2019 [32]	Korea	412	Male: 63.1 ± 10.7 Female: 65.9 ± 11.4	NRS 2002	<3 vs. 3 vs. ≥ 4	47.1 / 19.7 / 33.2	1.238	1.143–1.341	4.2	III-IV	Chemoradiotherapy / Surgery / No treatment	R	8
Rivelsrud 2021 [33]	Norway	149	66.6 ± 12.1	NRS 2002	<3 vs. ≥ 3	68.5	1.240	0.830–1.850	9	——	——	R	7
Trestini 2020 [34]	Italy	73	65 ± 11	NRS 2002	<3 vs. ≥ 3	80.8	5.240	1.420–19.320	3.7	I-IV	Surgery、Chemotherapy	R	8
Dang 2022 [35]	China	382	57.5(28.0–78.0)	COUNT	<2 vs. ≥ 2	73.0	1.145	1.051–1.248	3.4	I-IV	Surgery	P	8
				GPS	≤ 1 vs. >1	——	0.918	0.608–1.385					
Kato 2018 [36]	Japan	344	64.8 ± 9.9	CONUT	<4 vs. ≥ 4	23.0	1.640	1.190–2.260	14.9	I-IV	Surgery	R	8
Terasaki 2021 [20]	Japan	307	——	CONUT	≤ 3 vs. ≥ 4	9.1	1.750	1.010–3.050	5.2	I-IV	Surgery、chemotherapy	R	6
				PNI	<50 vs. ≥ 50	39.7	1.190	0.860–1.660					
				GPS	0 vs. ≥ 1	23.5	0.820	0.550–1.240					
Uemura 2022 [37]	Japan	110	66(38–84)	CONUT	0–1 vs. 2–4	57.2	1.920	1.160–3.240	5.2	——	chemotherapy	R	6
					0–1 vs. 5–8	7.2	10.710	3.870–27.630					
Wang 2020 [38]	China	294	55.5 ± 10.8	CONUT	<3 vs. ≥ 3	34.0	4.000	2.820–5.600	5.3	I-III	surgery	R	6
				PNI	<46.1 vs. ≥ 46.1	——	0.890	0.620–1.290					
Funamizu2022 [39]	Japan	139	——	GNRI	<99 vs. ≥ 99	51.8	2.490	1.370–4.540	5	I-IV	Surgery、chemotherapy	R	7
Hu 2020 [40]	China	282	58.7 ± 13.5	GNRI	≤ 98 vs. >98	36.9	1.757	1.318–2.341	9.6	I-IV	Surgery / chemotherapy	R	8
Kokumai 2021 [41]	Japan	41	65(41–79)	GPS	0 vs. 1–2	39.0	3.437	1.116–10.589	——	I-III	Conversion surgery	R	6
Bicakli 2020 [42]	Turkey	96	60.7(28–80)	PG-SGA	<9 vs. ≥ 9	85.5	4.660	1.650–13.190	2	——	Surgery / chemotherapy	P	7
Heckler 2021 [43]	Germany	116	65.1 ± 11.0	NRI	≤ 100 vs. >100	5.0	1.340	0.780–2.310	3	I-IV	Surgery / chemotherapy	P	8
				NRS2002	<3 vs. ≥ 3	78.0	1.450	0.810–2.590					
				SGA	A vs. B,C	37.0	2.170	1.370–3.470					
				MUST	0 vs. ≥ 1	54.0	1.360	0.850–2.210					
				MNA	<24 vs. ≥ 24	69.0	1.030	0.620–1.720					
				MNA-SF	≤ 11 vs. ≥ 12	92.0	0.530	0.250–1.110					
Nakagawa 2018 [44]	Japan	151	70(61—75)	PNI	<40.008 vs. ≥ 40.008	37.7	0.780	0.500–1.210	2.9	I-IV	Surgery、chemotherapy	R	8
Shirakawa 2023 [45]	Japan	255	65 (29–86)	PNI	< 47vs ≥ 47	—	1.440	0.890–2.340	—	IV	chemotherapy	P	6
Hayashi 2023 [46]	Japan	162	66.8 ± 8.9	PNI	< 45 vs. > 45	51.2	1.090	0.680–1.750	—	I-IV	Surgery、chemotherapy	R	7
Ma 2023 [47]	Canada	263	64 (19–84)	PNI	< 45 vs. ≥ 45	78.0	1.270	0.920–1.770		IV	chemotherapy	P	7

R, retrospective cohort study; P, prospective cohort study

In the 27 articles, 9 types of nutritional screening tools ( NRS2002 [32–34, 43], PNI [19, 20, 23–29, 31, 38, 44–47], GPS [19, 20, 23, 27, 31, 35, 41], mGPS [19, 24, 30, 31], CONUT [19, 20, 24, 35–38], MUST [43], MNA-SF [43], NRI [43] and GNRI [19, 39, 40]), 3 types of nutritional assessment tools(SGA [43], PG-SGA [42], MNA [43]) were reported in Table 2. The most used tool was PNI (*N* = 16), followed by CONUT(*N* = 7) and GPS(*N* = 7).

**Table 2:** Nutrition screening and assessment tools

Tool	Content									
	body mass index (BMI)	weight loss	acute disease effect (CRP)	serum albumin	total lymphocyte count.	Food intake	ability to eat	neuropsychological problems & stress factors& mobility	physical examination	Others
**Malnutrition risk screening tool**										
NRS-2002	√	√				√				disease severity, and age.
MUST	√	√	√							
MNA-SF	√	√					√	√		calf circumference.
PNI				√	√					
GPS			√	√						
mGPS			√	√						
NRI				√						the ratio of actual to usual weight.
CONUT				√	√					total cholesterol level.
GNRI				√						body weight, height.
**Malnutrition assessment tool**										
SGA		√							√	patient’s clinical history, changes in dietary.
PG-SGA		√				√				nutrition effect symptoms, activities and functions effected by nutrition. The professional component includes disease and age, the metabolic stress state, loss of subcutaneous fat, muscle wasting, edema and ascites.
MNA	√	√					√	√	√	place patient lives, medication, pressure sores or skin ulcers, number of meals, diet, mode of feeding, self view of nutritional and health status.

### Meta-analysis of the prognostic value of all-cause mortality in PC patients

Based on results from fixed-effects and random-effects models, poor nutritional status as determined by CONUT(HR = 1.978, 95%CI 1.345–2.907, *P* = 0.001), GNRI(HR = 1.595, 95%CI 1.033–2.466, *P* = 0.036), GPS(HR = 1.464, 95%CI 1.299–1.650, *P*<0.001), NRS2002(HR = 1.248, 95%CI 1.155–1.348, *P*<0.001) and PNI(HR = 1.504, 95%CI 1.295–1.747, *P*<0.001) was associated with mortality due to all causes in PC patients. As a result, mGPS (HR = 1.793, 95%CI 0.883–3.643, *P* = 0.106) was unable to show that abnormal nutritional status in PC patients was a significant predictor of all-cause mortality. According to Table 3, CONUT had the highest mortality rate among these tools.

**Table 3:** Analyses of all-cause mortality for PC patients

Tools	No. of studies	Heterogeneity		Model	Meta	
		I^2^(%)	Ph		HR(95%CI)	*P*
CONUT	7 [19, 20, 24, 35–38]	90.4	*P*<0.001	Random	1.978(1.345–2.907)	*P* = 0.001
GNRI	3 [19, 39, 40]	77.6	*P* = 0.012	Random	1.595(1.0332–2.466)	*P* = 0.036
GPS	7 [19, 20, 23, 27, 31, 35, 41]	48.3	*P* = 0.060	Fixed	1.464(1.299–1.650)	*P*<0.001
mGPS	4 [19, 24, 30, 31]	77.9	*P* = 0.004	Random	1.793(0.883–3.643)	*P* = 0.106
NRS2002	4 [32–34, 43]	39.2	*P* = 0.176	Fixed	1.248(1.155–1.348)	*P*<0.001
PNI	12 [19, 20, 23–29, 31, 38, 44–47]	60.6	*P* = 0.002	Random	1.504(1.295–1.747)	*P*<0.001

Subgroup analysis was conducted based on sample size, follow-up duration, cutoff value, treatment method, tumor stage and region for CONUT, GPS and PNI. In these three groups, there were no significant differences between the heterogeneity of each subgroup and the whole cohort based on the subgroup analysis. Ample size, follow-up duration, cutoff value, treatment method, and tumor stage and region are not related to heterogeneity of CONUT, GPS and PNI groups, as shown in Table 4.

**Table 4:** Subgroup analyses of all-cause mortality for PC patients

Subgroup	No. of studies	Heterogeneity		Meta	
		I^2^(%)	Ph	HR(95%CI)	P
**CONUT**					
Sample size<200	2 [24, 37]	82.7	*P* = 0.003	2.879(1.072–7.731)	*P* = 0.036
Sample size>200	5 [19, 20, 30, 35, 36]	92.4	*P*<0.001	1.722(1.116–2.659)	*P* = 0.014
Follow-up duration ≤ 5 years	3 [19, 24, 35]	0.0	*P* = 0.722	1.156(1.065–1.254)	*P* = 0.001
Follow-up duration > 5 years	4 [20, 36–38]	83.9	*P*<0.001	2.712(1.600-4.596)	*P*<0.001
Cut-off ≤ 3	5 [19, 24, 35, 37, 38]	92.2	*P*<0.001	1.727(1.063–2.806)	*P* = 0.027
Cut-off>3	3 [20, 36, 37]	84.4	*P* = 0.002	2.741(1.195–6.291)	*P* = 0.017
Surgery	4 [19, 35, 36, 38]	94.1	*P*<0.001	1.719(1.047–2.877)	*P* = 0.032
Other theory	3 [20, 24, 37]	75.4	*P* = 0.007	2.439(1.259–4.726)	*P* = 0.008
China	2 [35, 38]	97.9	*P*<0.001	2.116(0.621–7.207)	*P* = 0.231
Japan	5 [19, 20, 24, 36, 37]	72.4	*P* = 0.003	1.874(1.285–2.733)	*P* = 0.001
**GPS**					
Surgery	4 [19, 23, 31, 35]	31.1	*P* = 0.226	1.462(1.281–1.669)	*P*<0.001
Other theory	4 [20, 27, 31, 41]	67.3	*P* = 0.027	1.470(1.115–1.940)	*P* = 0.006
Sample size ≤ 200	4 [23, 27, 31, 41]	56.3	*P* = 0.076	1.693(1.405–2.040)	*P*<0.001
Sample size>200	4 [19, 20, 31, 35]	0.0	*P* = 0.440	1.323(1.132–1.545)	*P*<0.001
Cut-off = 1	5 [19, 20, 23, 31, 41]	11.9	*P* = 0.339	1.478(1.302–1.677)	*P*<0.001
Cut-off = 2	2 [27, 35]	86.9	*P* = 0.006	1.351(0.937–1.950)	*P* = 0.108
Advanced stage	5 [20, 27, 31, 35, 41]	62.4	*P* = 0.031	1.333(1.062–1.673)	*P* = 0.013
Early stage	3 [19, 23, 31]	0.0	*P* = 0.372	1.517(1.318–1.746)	*P*<0.001
**PNI**					
Cut-off ≤ 45	6 [23, 27, 29, 31, 44, 46, 47]	71.3	*P* = 0.001	1.740(1.550–1.950)	*P*<0.001
Cut-off>45	9 [19, 20, 24–26, 28, 38, 45]	0.0	*P* = 0.581	1.480(1.340–1.630)	*P*<0.001
Advanced stage	9 [20, 24, 26–29, 31, 44, 46]	71.4	*P*<0.001	1.430(1.170–1.750)	*P*<0.001
Early stage	8 [19, 23, 25, 28, 31, 38, 45, 47]	0.0	*P* = 0.798	1.520(1.340–1.710)	*P*<0.001
Surgery	5 [19, 23, 25, 31, 38]	76.3	*P* = 0.001	1.530(1.170–2.020)	*P* = 0.002
Other theory	11 [20, 24, 26–29, 31, 44–47]	0.0	*P* = 0.713	1.450(1.310–1.600)	*P*<0.001
Sample size ≤ 200	9 [23–25, 27–29, 31, 44, 46]	0.0	*P* = 0.814	1.460(1.270–1.670)	*P*<0.001
Sample size>200	8 [19, 20, 26, 28, 31, 38, 45, 47]	75.6	*P*<0.001	1.490(1.230–1.810)	*P*<0.001

### Sensitivity analyses and publication bias

We performed a sensitivity analysis in CONUT, GPS, mGPS, NRS2002, and PNI groups to determine whether omitting any study would affect the pooled HR. To assess publication bias, we also conducted Begg’s funnel plot and Egger’s linear regression test. As *P* = 0.266 for Begg’s test and *P* = 0.041 for Egger’s test in CONUT groups, *P* = 0.089 for Begg’s test and *P* = 0.036 for Egger’s test in mGPS groups and *P* = 0.669 for Begg’s test and *P* = 0.026 for Egger’s test in PNI groups indicted slight publication bias. Then trim and fill analysis showed robust results. As *P* = 0.711 for Begg’s test and *P* = 0.356 for Egger’s test in GPS group and *P* = 0.089 for Begg’s test and *P* = 0.255 for Egger’s test in NRS2002 groups showed no significant publication bias.

### Qualitative analysis of prognostic value in PC patients

The remaining 2 studies used PG-SGA, NRI, SGA, MUST, MNA and MNA-SF to evaluate PC patients’ nutritional status. Heckler reported that malnutrition assessed by NRI, MUST, MNA could not predict OS in 116 PC patients [43]. Nutritional screening tools MNA-SF and SGA and PG-SGA indicate that abnormal nutritional status is an important predictor of all-cause mortality in PC patients.

## Discussion

In this systematic review, we summarized the prognostic value of different nutritional screening and assessment tools for PC patients, including NRS2002, PNI, GPS, mGPS, CONUT, MUST, MNA-SF, NRI, GNRI, SGA, PG-SGA and MNA. Our study demonstrated that these tools exclude mGPS, NRI, MUST and MNA could predict survival of patients with pancreatic cancer. And CONUT had the maximum prognostic potential for mortality in PC patients.

The CONUT scoring system comprises of lymphocyte count, serum albumin, and total cholesterol. Serum albumin is a frequently utilized indicator for evaluating nutritional status, and numerous studies have demonstrated that low serum albumin levels are an autonomous predictor of poor survival for diverse cancers [11, 48]. Furthermore, total lymphocyte count associated with human nutrition [49]. While the PNI also incorporates serum albumin and peripheral blood lymphocytes, the primary distinction from CONUT is the lack of a total cholesterol calculation. According to Kheirouri [50], the CONUT score has demonstrated greater precision than the PNI in prognosticating survival across diverse cancer types, rendering it a more desirable tool. The cellular membrane represents a crucial constituent, wherein cholesterol serves not only as a marker of caloric consumption [51, 52], but also as a contributor to tumorigenesis and immune-related signaling pathways [53, 54]. Investigations have revealed that diminished levels of cholesterol are linked to inferior survival outcomes, plausibly due to the involvement of cholesterol in numerous biochemical pathways that underlie immune responses and tumor development [55]. . Existing studies have fully demonstrated that CONUT score is more effective than other prognostic score [56, 57], this is consistent with the conclusion of this study.

The NRS2002 tool demonstrated superior predictive ability for mortality risk in patients with PC (HR = 1.248, 95%CI (1.155–1.348), *P* < 0.001), despite its subjective nature, which requires patients to report recent changes in weight and eating habits. European Society for Parenteral and Enteral Nutrition (ESPEN) recommends the use of NRS2002 for both cancer and surgical patients [58, 59]. . Due to its ease of use and lack of reliance on laboratory indices, NRS2002 is frequently employed as a preoperative nutritional screening tool for cancer patients in certain Chinese medical institutions [60, 61]. .

In 2005, Bouillanne [62] established the GNRI as an objective and simple nutritional screening tool determined by serum albumin, height, and body weight. In elderly long-term care patients, it has been shown to be a useful tool in predicting mortality [63, 64]. Nurses only need to measure the patient’s weight and height, and take blood samples in a few minutes, which is less of a burden for older patients. Especially for some patients with cognitive, hearing and visual impairment, as well as some uncooperative patients, objective data can be used to better to evaluate the nutritional status of patients. According to Grinstead, monitoring nutritional status using weight and albumin to promote increased survival is vital to promoting survival after initial diagnosis [65].

C-reactive protein (CRP) and serum albumin were used in the GPS, which was proposed by McMillan [66] in 2013. CRP elevation indicates systematic inflammation [67, 68]. And it reflects growth activity in tumors, because tumors can produce cytokines, which increase the inflammatory response [69]. In addition, this scoring system can distinguish between different stages of cachexia [70]. . According to Yamada [71], GPS outperformed other inflammation-based markers in predicting survival in PC patients. Based on GPS, mGPS was modified with more detailed criteria. In a large patient cohort, Proctor [72] evidenced that the mGPS as the systemic inflammatory response, is a powerful prognostic factor compared with other biochemical parameters. Whereas, this study found no significant difference in mGPS’s predictive value in predicting the prognosis of PC patients (*P* = 0.106), which may be due to the limited literature included and the differences in treatment methods of study subjects affecting the results. More researches were needed in the future to verify mGPS’s prognostic effect of PC patients.

Our meta-analysis suggested that low PNI were associated with poor OS in patients with PC. And high CONUT, GPS, NRS2002 were correlated with worse OS in PC patients. Subgroup analysis based on sample size、follow-up duration、cutoff value、treatment method、tumor stage and country region also confirmed that PNI, CONUT, GPS, NRS2002 functioned as prognostic indicators for PC. The remaining studies used PG-SGA, NRI, SGA, MUST, MNA and MNA-SF to evaluate PC patients’ nutritional status. According to our qualitative studies, malnutrition assessed by NRI, MUST, and MNA could not predict all-cause mortality in PC patients. Other findings in PC patients indicated that abnormal nutritional status was an important determinant of survival. Nevertheless, nutritional status is not always a good predictor of all-cause mortality in PC patients [15]. Researchers found that different nutritional tools were significantly different in their predictive value for all-cause mortality in nine studies that used two or more nutritional tools simultaneously [19, 20, 23, 24, 27, 31, 35, 38, 43]. . Therefore, nutritional screening and assessment tools should be chosen based on the characteristics of PC patients and clinical settings.

To our knowledge, this study is the first attempt to evaluate the effect of nutritional screening and assessment tools on survival from pancreatic cancer. However, due to insufficient studies on nutritional assessment tools, only nutritional screening tools were meta-analyzed, and nutritional assessment tools were descriptive. And the current study has several potential limitations. First, we did not include ongoing studies and limited our search to English language publications. In addition, credible conclusion about the predictive value of these nutritional screening and assessment tools established on more studies was necessary because of the small sample size of the current meta-analysis. Second, the Newcastle-Ottawa Scale require judgment (i.e. subjective) and could differ across people. Third, this Meta-analysis indicated large heterogeneity in the predictive value of these tools. However, subgroup analysis failed to fully explain the cause of heterogeneity. Cancer stage、follow-up years and treatments were important confounding factors for OS, and not all the studies provide the information. Finally, most included studies were from China or Japan. A comprehensive and thorough investigation of the subject may be enhanced by gathering information from western countries.

## Conclusion

We found that poor nutritional status evaluated through GNRI, PNI, CONUT, NRS2002, and GPS significantly predicted mortality from all causes in PC patients. A nutritional screening tool with the highest predictive value was CONUT. Nutritional screening and assessment tools should be selected according to the purpose, the characteristics of the patient, and the clinical setting. To provide more tools for PC patients to predict their prognosis, large-scale studies are needed to prove the clinical application value of SGA, PG-SGA, and MNA-SF.

## Data Availability

The datasets used and/or analyzed during the current study are available from the corresponding author on reasonable request.
